# Imported Rabies, European Union and Switzerland, 2001–2010

**DOI:** 10.3201/eid1704.101154

**Published:** 2011-04

**Authors:** Nicholas Johnson, Conrad Freuling, Daniel Horton, Thomas Müller, Anthony R. Fooks

**Affiliations:** Author affiliations: Veterinary Laboratories Agency–Weybridge, Woodham Lane, UK (N. Johnson, D. Horton, A.R. Fooks);; Friedrich-Loeffler-Institute, Wusterhausen, Germany (C. Freuling, T. Müller)

**Keywords:** Viruses, rabies, zoonoses, European Union, Switzerland, letter

**To the Editor:** Europe is progressively becoming free of sylvatic rabies. However, reintroduction remains a threat. We report the incidence of rabies importation into the European Union (EU) and Switzerland and highlight common pathways for rabies introduction.

Rabies is a notifiable disease within the EU. Through comprehensive oral vaccination campaigns most EU member states have eliminated the disease. Despite this success, the danger of reintroduction of the disease is ever present. Rabies could be reintroduced through direct reentry of infected animals across land borders, as has happened in eastern Italy with an outbreak of fox rabies originating from the western Balkan region (*1*). Alternatively, reintroduction can occur through illegal or accidental import of an infected animal.

In an attempt to mitigate direct reentry, vaccination campaigns and technical assistance in nonmember states and in the EU have attempted to reduce the incidence of disease throughout the continent. Avoidance of importation of infected companion animals is achieved through enforcement of legislation. EU regulation No. 998/2003 defines the requirements that dogs or cats must meet before entry into the EU with the aim of preventing an infected but asymptomatic dog or cat from entering a member state from another country. Entry requirements include that the animal is identifiable by a microchip or tattoo, has been vaccinated against rabies, and, depending on the status of the country of origin according to Annex II of regulation No. 998/2003, has been serologically tested. A veterinary certificate should accompany the animal during the period of travel. Failure to meet these requirements could lead to the animal being returned to its country of origin, isolation of the animal until it meets the requirements, or, as a last resort if the first 2 options are not feasible, euthanasia of the animal. Certain member states also stipulate application of antiparasite treatments before entry.

Despite these regulations, importation of animals incubating rabies can still occur through failure of border controls, ignorance of importation rules, or active subversion of these rules. The Technical Appendix lists documented cases during 2001–2010 of rabid dogs brought into the EU. Control measures in the form of euthanizing animals and contact investigation have ensured that the disease did not become established in a carnivore reservoir and no human incidence of disease resulted. Vigilance at the level of veterinary practitioners has also enabled quick discovery of suspected animal cases, which limited the number of secondary animal cases (Technical Appendix). However, these discoveries point to a persistent trend of illegal animal movement into the EU.

Ignorance on the part of tourists of the danger of importing infectious disease and the rules governing animal movement underpin most of the cases. Juvenile dogs feature in many of the reports, presumably because puppies are attractive to tourists and, being small, are easily moved (*2**,**3*). A recurring pathway is that of vacationers visiting Morocco and returning to France through the Iberian Peninsula. This pathway has been confirmed in 4 cases and is suspected in several other instances (Technical Appendix; *4*). In addition, rabies in dogs has been reported in the Spanish enclaves of Ceuta and Melilla on the north coast of Africa in recent years (*5*). A further route of introduction appears to be from western Balkan countries into Germany (*6*).

The costs associated with such introductions are numerous. These costs include the diagnostic investigation of suspected cases, particularly if molecular analysis is required to confirm the source of the incursion, as was required when rabies was detected in a puppy in Switzerland (*7*).

Subsequent investigations to identify animal and human contact cases, often requiring >1 national or international agency, are needed to ensure that the disease has not spread and potential human contacts receive appropriate postexposure prophylaxis. In some cases the implementation of hotlines and several press releases was necessary to cope with the demand for information by the public (*4*). However, although media attention in such cases reached its primary and immediate objective, i.e., no secondary human rabies cases were reported, it may also have contributed to enhancing the sense of rabies risk, thereby prompting persons to associate dog bites in general with rabies and thus leading to increased numbers of persons seeking postexposure prophylaxis unnecessarily for several months (*8*). Further costs are also incurred in the euthanasia or quarantine of contact animals.

The evidence suggests that this trend for importation of animals incubating rabies will continue, requiring member states to maintain vigilance with measures appropriate to the potential risk and consequences of a rabies outbreak. This vigilance should involve rapid investigation of suspected cases of disease, maintenance of rabies diagnostic capacity and contingency plans, and improved coordination between member states to deal with disease introduction.

## Supplementary Material

Technical Appendix Table depicting documented cases of rabies imported into European Union member states and Switzerland, 2001-2010.
