# Supplementary material for: Imported Rabies, European Union and Switzerland, 2001–2010
Source: Emerg Infect Dis. 2011 Apr;17(4):751–3. doi: 10.3201/eid1704.101154 (PMC3377407; doi:10.3201/eid1704.101154)
Supplement: Technical Appendix — Table depicting documented cases of rabies imported into European Union member states and Switzerland, 2001-2010. [file 10-1154-Techapp.pdf]

Article DOI: 10.3201/eid1704.101154

Imported Rabies, European Union and Switzerland, 2001–2010

Technical Appendix

Technical Appendix Table. Documented cases of rabies imported into European Union member states and Switzerland, 2001–2010.

Year	Country	Source	Case details	Reference
2001	Germany	Nepal	Two German nationals acquired a dog in Nepal and subsequently had it vaccinated in Iran on January 16, 2001. Two to 3 days later, the dog began behaving aggressively and was euthanized on January 23. The cadaver was then driven back into Germany. Both men had received preexposure vaccination before travel and so received a single booster inoculation of rabies vaccine. The remains of the dog were tested and demonstrated to be rabies positive.	(*1*)
2001	France	Morocco	Two French nationals were on an extended holiday in Morocco traveling by camper van. On March 26, 2001, they adopted a 3-month-old puppy from a campsite near Agadir. They returned to France on March 31, traveling through Spain. On May 19, the health of the puppy declined, and it became aggressive within a day. On the basis of suspicion of rabies, the animal was euthanized, and test results were positive. An investigation confirmed contact with at least 5 humans and another dog while the 2 persons were in France. This animal was subsequently euthanized as a precautionary measure.	(*2*)
2002	Germany	Azerbaijan	A German national working in Baku, Azerbaijan, found a 1-month-old puppy in November 2001 and arranged for it to be vaccinated the same month. A veterinary inspection on December 18 confirmed the animal appeared healthy; 3 days later the animal was flown back to southern Germany. On December 23, the dog’s behavior changed, and it became anorexic. By January 7, 2002, nervous signs and aggression developed in the dog; it was euthanized January 9. A positive diagnosis of rabies was confirmed by the National Reference Laboratory. Six persons received postexposure prophylaxis as a result of contact with the dog.	(*3*)
2002*	France	Morocco	Rabies was reported in an imported dog.	
2003	Switzerland	North Africa	In May 2003, an abandoned puppy, estimated to be <1 month old, was brought to an animal shelter in Geneva. On June 25, the animal was adopted by a family in the Canton of Vaud. Less than 1 month later, the dog became progressively more aggressive and was euthanized July 19. Rabies was confirmed from brain samples; this case was determined to be an example of illegal importation into Switzerland.	(*4*)
2004	France	Morocco	A 4-month-old puppy imported into France July 11, 2004, became ill on August 18, and died August 21. Rabies was confirmed. The animal originated in Morocco and was transported to France through Spain by road. The puppy had traveled extensively in southeastern France with its owners, visiting several art festivals. Two further illegal imports of dogs were reported in France during 2004, both brought from Morocco through Spain.	(*5*)
2004*	France	Morocco	Rabies was reported in an imported dog.	
2004*	France	Morocco	Rabies was reported in an imported dog.	
2004*	Germany	Morocco	On January 6, 2004, an 8-month-old dog was confined at the airport in Hannover because of absence of import documentation and no evidence of antirabies vaccination. The dog had been transported in hand luggage and had had a stopover in Nuremberg without customs control. Import permission was requested and granted under the condition of immediate vaccination and quarantine at the owners’ premises. However, because of enteritis, the vaccination was postponed by the veterinary officer. On February 2, hind leg paresis developed in the dog and rabies was suspected. The dog died 5 days later in quarantine and rabies was confirmed by laboratory testing. Epidemiologic investigations identified 20 people at risk for exposure, and all received postexposure prophylaxis. One cat with direct contact with the dog was euthanized, and 2 dogs with possible contact were vaccinated.	

2007†	Finland	India	In November 2007, a puppy was imported into Finland from India by a private owner. The puppy was vaccinated against rabies <30 days before import, but the vaccination date was not entered correctly into the official certificate, and no antibody test was conducted. The puppy was supervised by veterinary officers starting on November 5 because of deficiencies in the health certificates. It became sick on November 8 (fever and apathy) and was euthanized November 9. The cadaver was sent to the Finnish Food Safety Authority for pathologic analysis and tested for rabies. Rabies infection was confirmed November 14; the virus was 98% identical to Indian rabies virus strains. No animal contacts were identified.	
2007	Belgium	Morocco	During autumn 2007, a 4–5-week-old puppy was illegally brought into Belgium by air smuggled in a handbag. No vaccination was sought before departure or on arrival in Belgium. The puppy exhibited behavioral changes on October 16; neurologic signs developed in the puppy November 19. The puppy was euthanized a day later. The cadaver was submitted to the National Reference Laboratory for Rabies, and a positive diagnosis was made. A second dog owned by the family was also euthanized.	(*6*)
2008	France	Morocco	On February 26, 2008, the National Reference Centre for Rabies confirmed rabies in a domestic dog from the outskirts of Paris. This animal had never traveled outside France. A follow-up investigation identified contact with a dog that had been euthanized on January 5 after a short unspecified illness. This second dog had in turn been in contact with a third dog that died in November 2007 of an illness that in retrospect could have been rabies. Investigation of the third dog showed that it had been imported illegally from Morocco and reached France by road after traveling through Portugal and Spain. Contact tracing identified 177 persons, of whom 152 received postexposure vaccination.Seven dogs and 1 cat were euthanized; all were rabies negative.	(*7*)
2008	Belgium/ France	Gambia	A Belgian national found an injured young dog on April 1, 2008, in the Gambia. The dog was adopted and received rabies vaccination on April 5, when it also was microchipped and given a certificate of good health. On April 7, the owner returned with the dog by plane directly to Belgium, where it stayed until April 13. The owner then traveled to France, where on April 16, vomiting and anorexia developed in the dog. After 3 veterinary inspections, rabies was suspected. The dog died April 21, and a positive laboratory test result confirmed the diagnosis. Subsequent investigations were conducted in France and Belgium.	(*8*)
2008	United Kingdom	Sri Lanka	A 10-week-old puppy was legally imported on April 17, 2008, as part of a single consignment of 13 animals rescued by an animal charity dedicated to rehoming stray dogs in Sri Lanka. It was housed in a quarantine facility in North London but died on April 25 after a short diarrheal illness and convulsions. Rabies was confirmed shortly afterward. This case was confirmed before the animal left the quarantine facility, the rabies-free status of the United Kingdom was not affected.	(*9*)
2008†	France	Morocco	On November 14, 2008, the Pasteur Institute confirmed rabies in a 3-month-old German Shepherd from Saint André-le-Gaz, in the Isère Department. The dog had died November 10 with neurologic signs, which had first developed on October 30. The dog was originally found in a car park in Malaga, Spain, on October 12 and was driven to France the same day. Once in France, the dog was confined to the new owners' premises. French authorities identified 30 persons who had contact with the dog during the infectious period.	
2008	Germany	Croatia	A 6-week-old puppy was transported in July from Croatia to an animal shelter in Germany. The owner indicated that there was no border control at the European Union border. The animal was euthanized December 29, 2008, because of suspicion of rabies. Rabies was confirmed. Contact investigation identified 27 persons who had possible contact with the dog; 9 received postexposure prophylaxis.	(*10*)
2009*	France	Afghanistan	Rabies was reported in an imported dog.	

2010*	Germany	Bosnia-Herzegovina	In Neustadt an der Aisch, an 8-week-old puppy imported from Bosnia-Herzegovina by its owner was euthanized and showed positive test results for rabies by the Bavarian Health and Food Safety Authority. Rabies was confirmed at the National Reference Laboratory at the Freidrich-Loeffler-Institut. The puppy had a history of being bitten by a suspected rabid dog in the country of origin. After arriving in Germany, clinical signs indicative of rabies, including hypersensitivity, aggression, increased salivation, and paresis, developed in the puppy. The puppy also bit the owner. Epidemiologic investigations identified contact with 2 other dogs; both had received vaccination against rabies. A total of 17 persons were given adequate antirabies postexposure prophylaxis.	

*Case reported to Rabies Bulletin Europe.
†Case reported to World Organisation for Animal Health but not elsewhere.

References

1. Döller PC. Case history: human exposure in connection with an Asian trip. Rabies Bulletin Europe. 2001;25.1:9–10.

2. Bruyere-Masson V, Barrat J, Cliquet F, Rotivel Y, Bourhy H, Brie P, et al. A puppy illegally imported from Morocco brings rabies to France. Rabies Bulletin Europe. 2001;25.3:12–3.

3. Suess WH, Berg B, Keller B, Schmahl W. Rabies in a vaccinated dog imported from Azerbaijan to Germany. Rabies Bulletin Europe. 2001;25.4:14–5.

4. Zanoni R, Breitenmoser U. Rabies in a puppy in Nyon, Switzerland. Rabies Bulletin Europe. 2003;27.3:5–7.

5. Servas V, Mailles A, Neau D, Castor C, Manetti A, Fouquet E, et al. An imported case of canine rabies in Aquitaine: investigation and management of the contacts at risk, August 2004–March 2005. Euro Surveill. 2005;10:222–5 PubMed

6. Le Roux I, Van Gucht S. Two cases of imported canine rabies in the Brussels area within six months time. Rabies Bulletin Europe. 2008;32.1:5–6.

7. French Multidisciplinary Investigation Team. Identification of a rabid dog in France illegally introduced from Morocco. Euro Surveill. 2008;13:pii: 8066. PubMed

8. French and Belgian Multidisciplinary Investigation Teams. Identification of a rabid dog illegally introduced from the Republic of the Gambia to Belgium and France. Euro Surveill. 2008;13:pii:8856. PubMed

9. Fooks AR, Harkess G, Goddard T, Marston DA, McElhinney LM, Brown K, et al. Rabies in a dog imported to the UK from Sri Lanka. Vet Rec. 2008;162:598. PubMed

10. Weiss B, Hoffmann U, Freuling C, Müller T, Fesseler M, Renner C. Rabies exposure due to an illegally imported dog in Germany. Rabies Bulletin Europe. 2009;33.2:5–7.
